# Quantity down, quality preserved: a continuous HOMA-IR analysis of IVF/ICSI outcomes in PMOS patients

**DOI:** 10.3389/fendo.2026.1930083

**Published:** 2026-09-23

**Authors:** Huiyi Xie, Jingu Li, Yunni Cai, Xiaoli Chen

**Affiliations:** Department of Reproductive Medicine, Quanzhou Women and Children’s Hospital (Quanzhou Children’s Hospital), Quanzhou, Fujian, China

**Keywords:** dose–response relationship, embryo developmental competence, HOMA-IR, implantation, insulin resistance, oocyte yield, PMOS, pregnancy loss

## Abstract

**Objective:**

To evaluate the dose-response relationship between HOMA-IR as a continuous variable and embryo development outcomes in fresh IVF/ICSI cycles of PMOS patients (formerly PCOS), distinguishing IR effects on oocyte yield (ovarian responsiveness) from effects on embryo developmental efficiency (per-oocyte conversion potential), and to evaluate differential IR impacts on cumulative implantation versus pregnancy maintenance.

**Design:**

Single-center retrospective cohort study of all fresh IVF/ICSI cycles from July 2022 to March 2026, comprising 3058 de-duplicated patients (2799 non-PMOS, 259 PMOS; PMOS-nonIR n=115, PMOS-IR n=144, cut-off ≥2.5). Spearman correlation and multivariable linear regression (adjusted for age, BMI, AMH) were used to evaluate the independent associations of HOMA-IR with oocyte maturation, fertilization, and embryo development at each stage.

**Results:**

Oocyte maturation rate showed no correlation with HOMA-IR (r=-0.014, *P* = 0.8178). HOMA-IR was negatively correlated with oocyte count (r=-0.226, *P* = 0.0002), and oocyte yield was significantly lower in PMOS-IR vs PMOS-nonIR. Transferable embryo and usable blastocyst counts were negatively associated with HOMA-IR, but transferable embryo rate showed no association with HOMA-IR (adjusted β=0.817, *P* = 0.3940), nor did blastocyst formation rate or usable blastocyst rate. Cumulative β-hCG positive and clinical pregnancy rates were significantly higher in PMOS-IR (85.4% and 81.9%) versus Non-PMOS (72.8% and 69.0%, *P* = 0.0024 and *P* = 0.0022), largely driven by greater transfer opportunity (97.9% vs 89.0%). By contrast, clinical pregnancy loss (no live birth among clinical pregnancies with a known outcome) showed a graded upward trend (13.5% vs 15.6% vs 17.2%), but the between-group difference was not statistically significant; owing to the limited number of loss events in the PMOS subgroups, this comparison should be interpreted with caution. Cumulative live birth rates did not differ significantly across groups (56.9% vs 56.5% vs 54.5%, *P* = 0.7853).

**Conclusions:**

In PMOS patients, IR exhibits multi-stage differential effects — impairing oocyte yield while preserving embryo developmental competence. Once oocytes are retrieved, their developmental efficiency is unaffected by IR status. Higher cumulative implantation in PMOS-IR was largely driven by greater transfer opportunity, whereas clinical pregnancy loss showed an upward trend without statistical significance, indicating that IR-related reproductive impairment primarily involves oocyte yield and transfer opportunity, while post-implantation pregnancy maintenance merits further evaluation. This warrants prospective validation. HOMA-IR should be integrated into pre-IVF ovarian response stratification but not used to predict embryo developmental competence.

## Introduction

Polyendocrine metabolic ovarian syndrome (PMOS), formerly polycystic ovary syndrome (PCOS), affects 8–13% of women of reproductive age (1). In May 2026, The Lancet published a global consensus formally renaming the condition (2); we adopt the new nomenclature throughout this paper. Insulin resistance (IR) is a core pathophysiological feature of PMOS, present in approximately 50–70% of patients (3, 4). HOMA-IR is a validated surrogate measure of IR (5), with a commonly used cut-off of ≥2.5 in Asian populations (6–8).

Although the clinical importance of IR is well established, its independent effect on IVF/ICSI embryo development outcomes remains controversial (9–13). A 2024 meta-analysis reported fewer mature (MII) oocytes and embryos, lower clinical pregnancy, and higher miscarriage (13). A 2025 cohort of 1,768 PMOS-IVF cycles demonstrated that IR reduces fertilization and 2PN rates (9); Wu et al. reported HOMA-IR as an independent negative predictor of live birth (14); a recent series of studies published in Frontiers in Endocrinology further confirmed the association between IR and PMOS-IVF outcomes (15, 16). However, most prior studies dichotomized HOMA-IR using an arbitrary threshold, discarding dose–response information and conflating oocyte yield (ovarian responsiveness) with embryo developmental efficiency (conversion potential per oocyte). Analyzing HOMA-IR as a continuous variable captures the full spectrum from insulin-sensitive to severely insulin-resistant states, with greater statistical power (17). Furthermore, whether IR affects embryo development indirectly through impaired oocyte maturation or directly at fertilization and post-fertilization stages remains insufficiently characterized in the existing evidence (9, 18).

Using continuous HOMA-IR data from 259 PMOS patients at our center, we applied rigorous rate definitions — transferable embryo rate = transferable embryos/normally cleaved oocytes; usable blastocyst rate = usable blastocysts/blastocysts formed — to disentangle IR effects on ovarian responsiveness from effects on embryo developmental efficiency. The key innovations of this study are (1): treating HOMA-IR as a continuous rather than binary variable, preserving full dose–response information (2); using rigorously defined rate metrics that separate quantitative oocyte yield from qualitative developmental efficiency (3); simultaneously evaluating three sequential stages — oocyte maturation, fertilization efficiency, and embryo development — to provide mechanistic localization of IR effects.

## Methods

### Study population

Single-center retrospective cohort study including all fresh IVF/ICSI cycles from July 2022 to March 2026. The analysis dataset comprised 3058 de-duplicated patients (2799 non-PMOS, 259 PMOS). PMOS was diagnosed according to the Rotterdam 2003 criteria (19). Only the first PMOS cycle per patient was retained to ensure statistical independence. Of note, fasting insulin is not a routine pre-IVF test at our center: only 435 of 2799 Non-PMOS patients (15.5%) had both fasting insulin and glucose measured — some records were simply incomplete in the electronic medical record system, and some were tested because clinicians suspected PMOS (but the patients ultimately did not meet Rotterdam criteria and were classified as Non-PMOS). Consequently, the Non-PMOS group’s metabolic profile is not fully representative of the general Non-PMOS population and should be interpreted with caution. Critically, the core analysis (HOMA-IR dose–response) is conducted entirely within the PMOS group and does not rely on Non-PMOS metabolic data. Stimulation protocol data were extracted from electronic medical records and classified into four groups: GnRH-a (including long, super-long, and modified-super-long protocols), Antagonist, PPOS, and Microstimulation (including natural cycles and ovulation induction). At our center, hCG (recombinant hCG 250 μg or urinary hCG 10,000 IU) is the standard final maturation trigger for >95% of IVF/ICSI cycles; GnRH-agonist trigger is reserved for high OHSS-risk cases. The study flow is shown in **Figure 1**.

**Figure 1:**
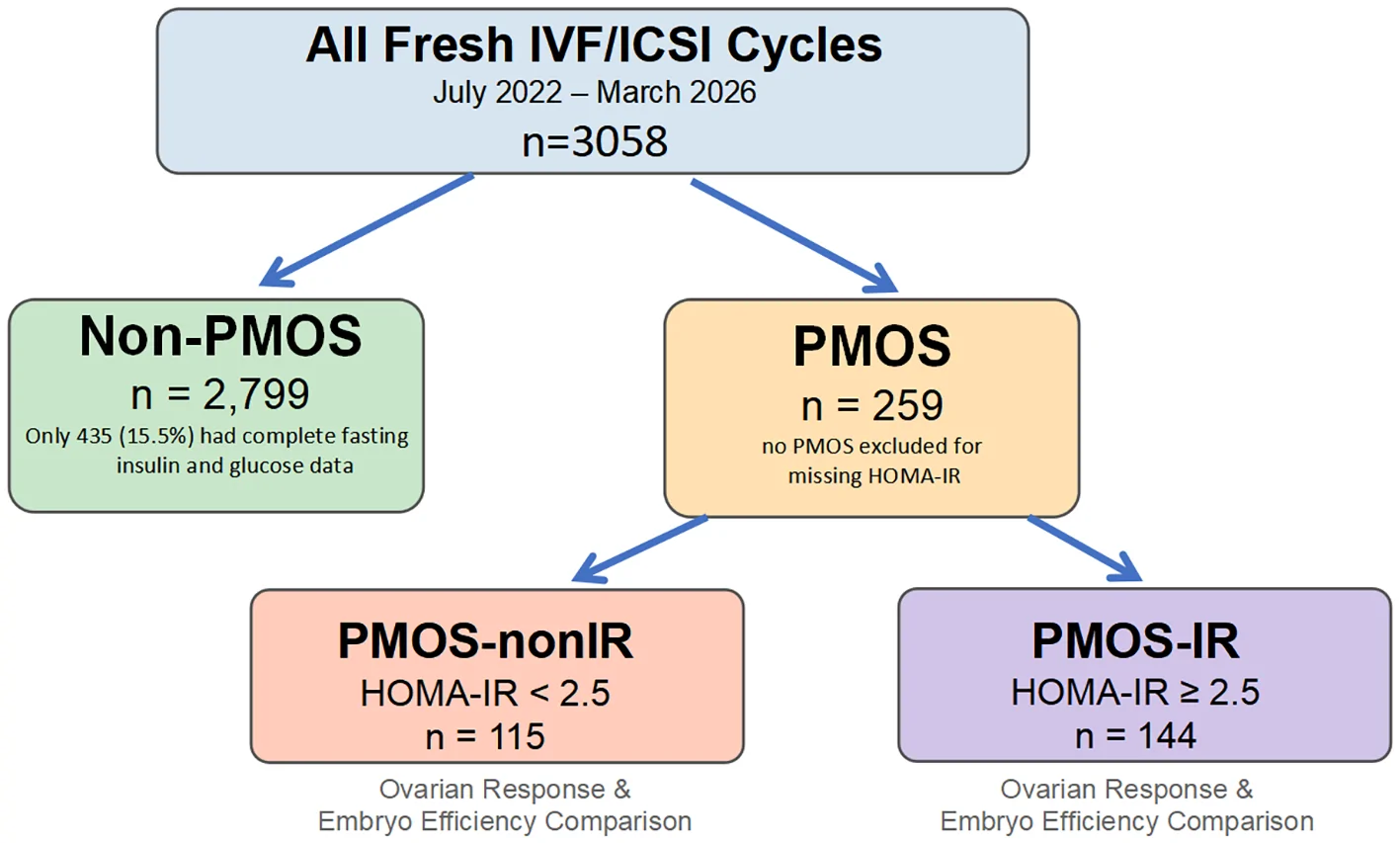
Study flow chart. PMOS = polyendocrine metabolic ovarian syndrome (formerly PCOS), renamed per the 2026 Lancet global consensus.

### Metabolic assessment

HOMA-IR = fasting insulin × fasting glucose/22.5 (5). For descriptive comparisons, IR was defined as HOMA-IR ≥2.5. Primary analyses treated HOMA-IR as a continuous variable. Continuous variables were Winsorized at 1%/99%.

### Outcome measures

We applied rigorous rate definitions: maturation rate = MII oocytes/oocytes retrieved × 100%; fertilization rate = fertilized oocytes/oocytes retrieved × 100%; 2PN rate = normally fertilized (2PN) oocytes/oocytes retrieved × 100%; transferable embryo rate = transferable embryos/normally cleaved oocytes × 100%; blastocyst formation rate = blastocysts formed/blastocysts cultured × 100%; usable blastocyst rate = usable blastocysts/blastocysts formed × 100%. Secondary clinical outcomes were assessed with all oocyte retrieval cycles in each group as the denominator (one cycle per patient, integrating data from fresh and subsequent FET cycles). β-hCG positivity was determined by laboratory assay. Clinical pregnancy was determined by pregnancy type. Live birth was defined as ≥1 live-born infant in the neonatal record or a recorded delivery date. Pregnancy loss was defined as no live birth but a recorded miscarriage, ectopic pregnancy, stillbirth, or induction of labor; pregnancies with neither a live birth nor a loss record (ongoing pregnancy or loss to follow-up) were excluded from the pregnancy-loss denominator.

### Statistical analysis

Spearman rank correlation and multivariable linear regression (adjusted for age, BMI, AMH) were performed. As a sensitivity analysis, we added protocol group dummies (Antagonist, PPOS; reference = GnRH-a) to the multivariable regression models to assess whether stimulation protocol confounded the HOMA-IR–outcome associations (207 patients with complete covariate data). Python 3.12 (scikit-learn, scipy, pandas). Two-sided *P* < 0.05 was considered statistically significant.

## Results

### Study population

The analysis dataset comprised 3058 de-duplicated patients (2799 non-PMOS, 259 PMOS; PMOS-nonIR n=115, PMOS-IR n=144). Baseline characteristics appear in **Table 1**: the PMOS-IR group had significantly higher BMI (26.04 ± 3.64 vs 22.53 ± 3.13 kg/m²) and ALT (27.03 ± 15.43 vs 19.55 ± 10.94 U/L) (both *P* < 0.001), suggesting that IR may co-occur with hepatic steatosis (20).

**Table 1:** Baseline characteristics by group.

Variable	Non-PMOS (n=2799)	PMOS-nonIR (n=115)	PMOS-IR (n=144)	_P_
Age (years)	33.08 ± 4.51	30.34 ± 3.86	30.81 ± 3.97	<0.0001
BMI (kg/m²)	22.53 ± 3.13	22.16 ± 2.96	26.04 ± 3.64	<0.0001
Fasting glucose (mmol/L)	4.90 ± 0.51	4.73 ± 0.38	5.12 ± 0.54	<0.0001
Fasting insulin (μIU/mL)	11.00 ± 6.79	7.48 ± 2.50	20.13 ± 8.12	<0.0001
HOMA-IR	2.51 ± 1.99	1.57 ± 0.51	4.66 ± 2.22	<0.0001
AMH (ng/mL)	3.21 ± 2.34	7.37 ± 3.57	7.21 ± 3.81	<0.0001
FSH (mIU/mL)	7.68 ± 3.46	6.48 ± 2.04	6.08 ± 1.63	<0.0001
LH (mIU/mL)	4.41 ± 3.19	7.83 ± 5.27	6.97 ± 6.35	<0.0001
E2 (pg/mL)	69.26 ± 120.02	67.02 ± 125.09	57.53 ± 62.77	0.5879
PRL (ng/mL)	14.14 ± 7.65	13.50 ± 7.92	12.69 ± 6.47	0.0491
TSH (mIU/L)	2.05 ± 1.13	1.94 ± 1.15	2.15 ± 1.19	0.2666
HDL (mg/dL)	59.38 ± 9.79	58.46 ± 10.04	59.58 ± 9.18	0.4391
LDL (mg/dL)	31.70 ± 7.91	33.07 ± 8.13	32.51 ± 7.82	0.0861
ALT (U/L)	19.55 ± 10.94	21.13 ± 12.62	27.03 ± 15.43	<0.0001
AST (U/L)	22.86 ± 8.06	24.14 ± 7.96	25.33 ± 8.49	0.0004

Data are mean ± SD. P values by Kruskal-Wallis test. In the Non-PMOS group, only 435 patients (15.5%) had both fasting insulin and glucose measured; insulin, glucose, and HOMA-IR for this group are reported from this subset. Both PMOS groups had complete data (100%).

As shown in **Table 2**, the PMOS-IR group required higher total gonadotropin doses and longer stimulation duration than the PMOS-nonIR group, indicating reduced ovarian responsiveness to exogenous gonadotropins. Stimulation protocol distribution among PMOS patients: GnRH-a = 167 (64.5%), Antagonist = 63 (24.3%), PPOS = 29 (11.2%). The protocol-adjusted sensitivity analysis (n=207) yielded β coefficients virtually identical to the primary model (e.g., oocyte count: β = -0.599 vs -0.605; transferable embryo count: β = -0.444 vs -0.441; transferable embryo rate remained non-significant: β = 0.701, *P* = 0.4635), confirming that protocol type does not confound the HOMA-IR–outcome associations.

**Table 2:** Ovarian response parameters.

Variable	Non-PMOS (n=2799)	PMOS-nonIR (n=115)	PMOS-IR (n=144)	_P_
Total Gn dose (IU)	2655.07 ± 922.82	2407.90 ± 852.54	3116.85 ± 1148.10	<0.0001
Gn duration (days)	11.27 ± 2.54	12.34 ± 2.67	13.67 ± 3.10	<0.0001
E2 on hCG day (pg/mL)	3013.34 ± 1976.69	4467.78 ± 2440.68	3401.44 ± 2102.96	<0.0001
Endometrial thickness (mm)	9.52 ± 2.70	10.11 ± 2.14	10.24 ± 2.20	0.0019
Follicles on hCG day	13.20 ± 6.76	20.05 ± 5.90	19.08 ± 6.36	<0.0001
Oocytes retrieved	10.88 ± 6.86	16.34 ± 7.76	13.62 ± 6.95	<0.0001

Data are mean ± SD. P values by Kruskal-Wallis test.

**Supplementary Figure S1** displays metabolic profiles by group (BMI, fasting glucose/insulin, HOMA-IR, HDL, LDL, ALT, AST) as box plots; BMI and ALT were significantly elevated in PMOS-IR.

### Oocyte maturation and fertilization

Oocyte yield was significantly lower in PMOS-IR versus PMOS-nonIR (13.62 ± 6.95 vs 16.34 ± 7.76, *P* < 0.0001), despite higher total Gn dose and longer stimulation in the IR group, indicating reduced ovarian responsiveness to exogenous gonadotropins. Maturation rate showed no difference across the three groups (80.59 ± 17.77% vs 80.49 ± 17.13% vs 80.29 ± 20.76%, *P* = 0.5173) and showed no correlation with HOMA-IR (r=-0.014, *P* = 0.8178), demonstrating that IR does not impair meiotic maturation. Fertilization rate was slightly lower in PMOS-IR than in PMOS-nonIR (72.33 ± 21.05% vs 75.03 ± 18.38%), reaching nominal significance across groups (*P* = 0.0275), whereas 2PN rate did not differ significantly (57.67 ± 21.08% vs 61.40 ± 17.80%, *P* = 0.0748). Despite this mild fertilization impairment, downstream embryo developmental efficiency was preserved. See **Table 3** for details. The between-group distribution of each metric is shown in **Supplementary Figure S2**.

**Table 3:** Embryo development outcomes.

Variable	Non-PMOS (n=2799)	PMOS-nonIR (n=115)	PMOS-IR (n=144)	_P_
Oocytes retrieved	10.88 ± 6.86	16.34 ± 7.76	13.62 ± 6.95	<0.0001
MII oocytes	8.56 ± 5.71	13.15 ± 6.93	11.08 ± 6.15	<0.0001
Maturation rate (%)	80.29 ± 20.76	80.49 ± 17.13	80.59 ± 17.77	0.5173
Fertilization rate (%)	75.71 ± 22.50	75.03 ± 18.38	72.33 ± 21.05	0.0275
2PN rate (%)	61.05 ± 24.37	61.40 ± 17.80	57.67 ± 21.08	0.0748
Transferable embryos	4.25 ± 3.59	6.81 ± 4.87	5.10 ± 3.61	<0.0001
Transferable rate (%)	67.59 ± 31.01	65.92 ± 28.65	68.14 ± 27.92	0.4961
Usable blastocysts	2.18 ± 2.30	3.71 ± 3.13	2.68 ± 2.55	<0.0001
Blastocyst formation rate (%)	54.60 ± 32.22	52.55 ± 29.13	52.76 ± 30.92	0.5835
Usable blastocyst rate (%)	70.33 ± 31.50	70.15 ± 24.70	68.60 ± 30.58	0.4039

Transferable rate = transferable embryos/normally cleaved oocytes × 100%. Usable blastocyst rate = usable blastocysts/blastocysts formed × 100%. P values by Kruskal-Wallis test.

### Dose–response: oocyte yield vs embryo efficiency

In the PMOS subgroup (n=259), HOMA-IR was negatively correlated with oocyte yield (r=-0.226, *P* = 0.0002; **Figure 2A**). After adjustment for age, BMI, and AMH, each 1-unit increase in HOMA-IR was associated with approximately 0.605 fewer oocytes retrieved (β=-0.605, *P* = 0.0128); it should be noted that PMOS patients overall had higher oocyte yield than Non-PMOS controls, so this β reflects a dose–response gradient of declining oocyte yield from a higher baseline within the PMOS subgroup as IR worsens. However, once oocytes were retrieved, embryo developmental efficiency showed no association with HOMA-IR (**Figures 2B–F**): transferable embryo rate (transferable embryos/normally cleaved oocytes) r=0.042, *P* = 0.5082; blastocyst formation rate r=-0.036, *P* = 0.5673; usable blastocyst rate r=0.037, *P* = 0.5874.

**Figure 2:**
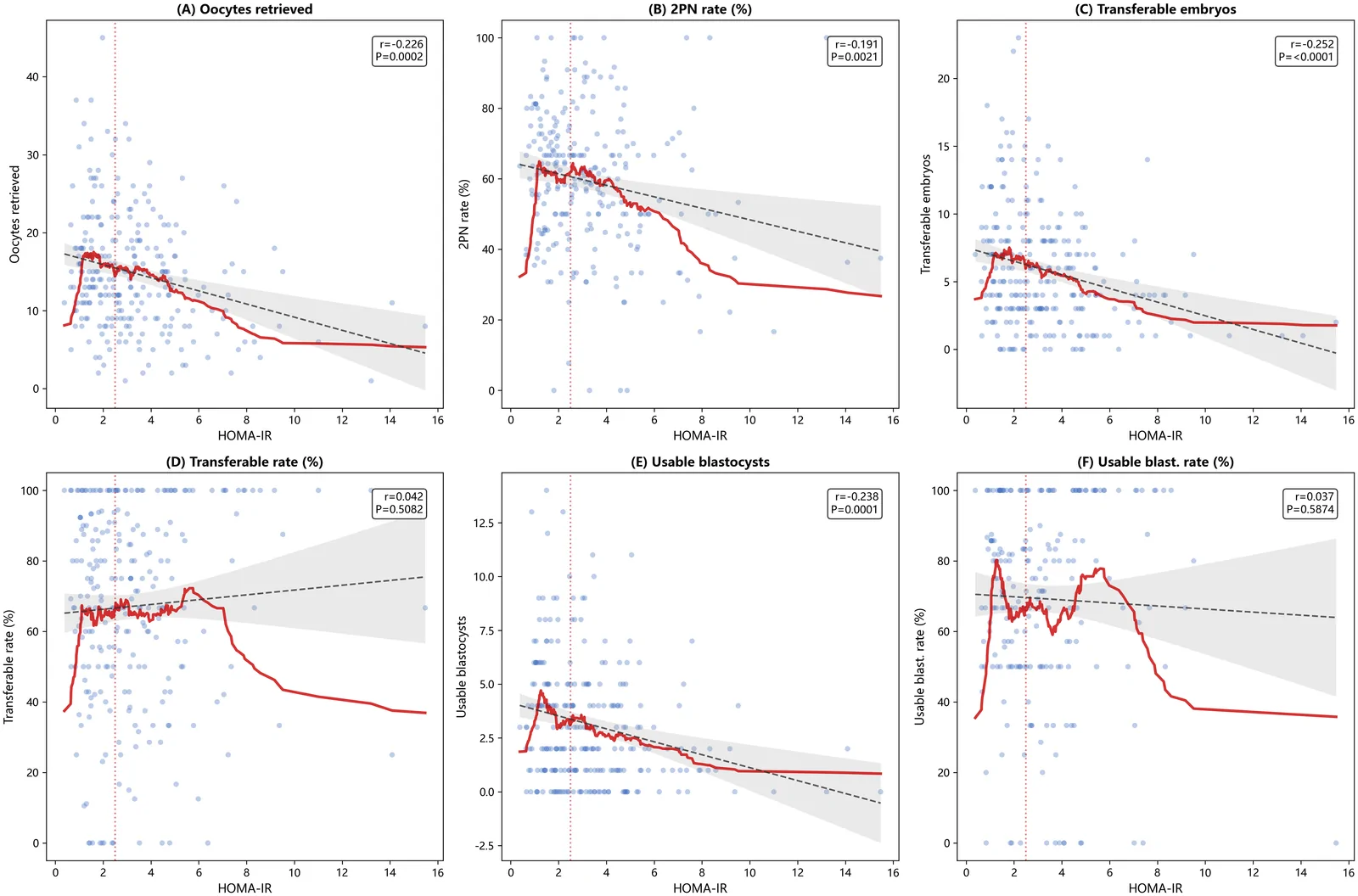
Dose–response relationships between HOMA-IR and oocyte yield and key reproductive outcomes. Red dashed line: HOMA-IR = 2.5 (IR cut-off). Red solid line: moving-average smooth. Black dashed line: linear fit. Gray shaded band: 95% confidence interval of the linear regression. Marker transparency reduced to mitigate visual overlap.

The per-oocyte conversion efficiency further illustrates this dissociation: the oocyte-to-transferable conversion efficiency was 41.7% in PMOS-nonIR vs 37.4% in PMOS-IR; the normally cleaved to transferable conversion efficiency was nearly identical (68.8% vs 67.4%). Multivariable linear regression further confirmed that, after adjustment, HOMA-IR was not associated with transferable embryo rate (β=0.817, *P* = 0.3940), but remained significantly associated with the absolute number of transferable embryos (β=-0.441, *P* = 0.0016; **Figure 2C**), confirming that the reduction in embryo numbers is driven by reduced oocyte yield rather than impaired developmental efficiency. The complete correlation matrix is shown in **Supplementary Table S1**, the full regression output in **Supplementary Table S2**, and the correlation heatmap in **Figure 3**.

**Figure 3:**
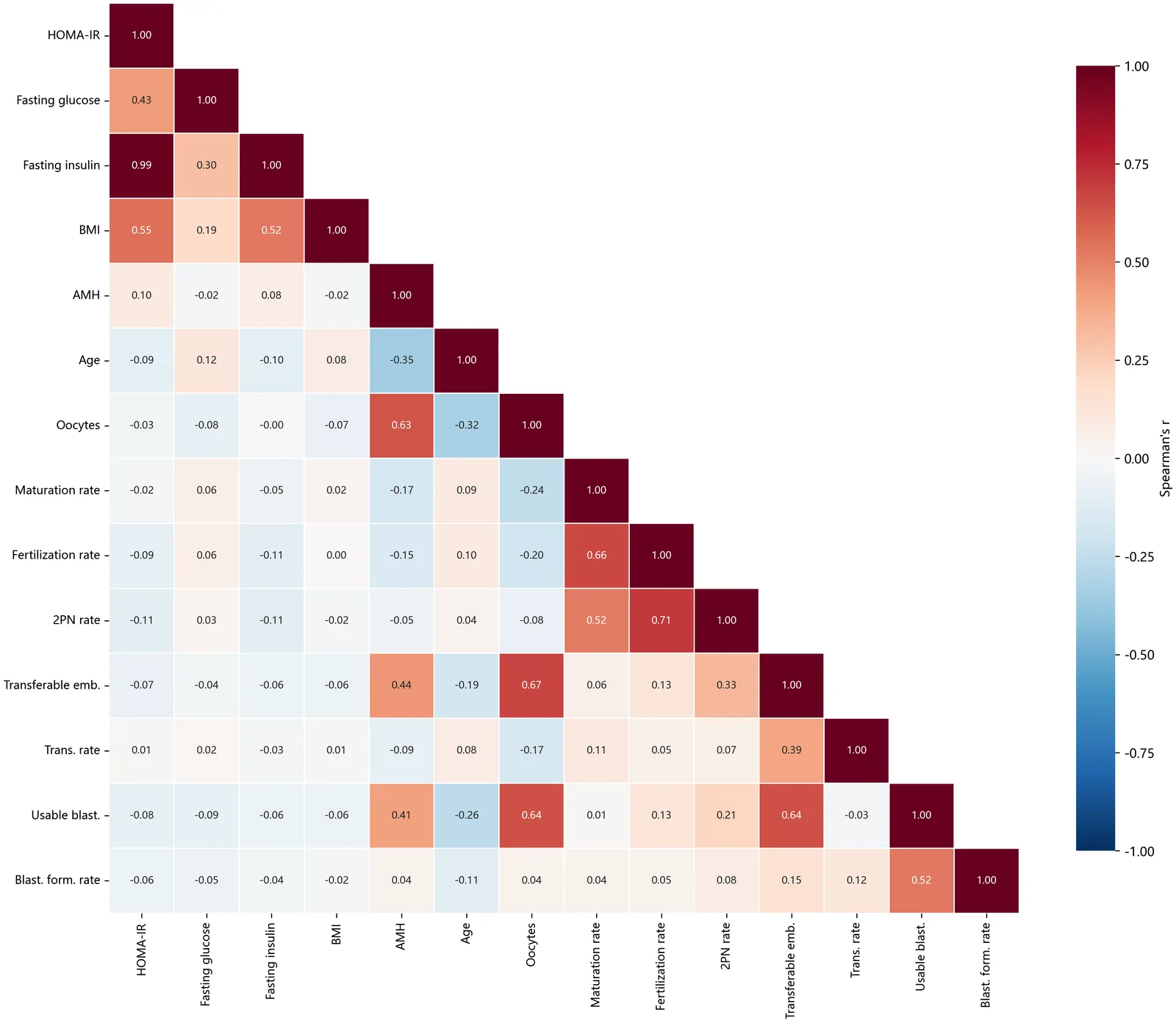
Spearman correlation heatmap of metabolic and reproductive variables in PMOS patients. Color scale represents Spearman correlation coefficient. Only the lower triangle is displayed.

### Clinical outcomes

**Table 4** presents cumulative clinical outcomes, with each group’s total oocyte retrieval cycles as the denominator (one cycle per patient, integrating fresh and subsequent FET cycles). Of 2799 non-PMOS patients, 2492 (89.0%) underwent ≥1 embryo transfer, yielding cumulative β-hCG positive, clinical pregnancy, and live birth rates of 72.8%, 69.0%, and 54.5%, respectively. The corresponding rates were 77.4%, 74.8%, and 56.5% in the PMOS-nonIR group (110/115, 95.7% with ≥1 transfer), and 85.4%, 81.9%, and 56.9% in the PMOS-IR group (141/144, 97.9% with ≥1 transfer). Cumulative β-hCG positive and clinical pregnancy rates were significantly higher in PMOS-IR versus non-PMOS (*P* = 0.0024 and *P* = 0.0022, respectively), but this advantage was largely driven by greater transfer opportunity (97.9% vs 89.0%), as per-transfer implantation rates did not differ significantly between groups.

**Table 4:** Cumulative clinical outcomes by group.

Outcome	Non-PMOS (n=2799)	PMOS-nonIR (n=115)	PMOS-IR (n=144)	_P_
β-hCG positive rate	72.8 ± 0.8% (2038/2799)	77.4 ± 3.9% (89/115)	85.4 ± 2.9% (123/144)	0.0024
Clinical pregnancy rate	69.0 ± 0.9% (1932/2799)	74.8 ± 4.0% (86/115)	81.9 ± 3.2% (118/144)	0.0022
Live birth rate	54.5 ± 0.9% (1526/2799)	56.5 ± 4.6% (65/115)	56.9 ± 4.1% (82/144)	0.7853
Pregnancy loss rate	13.5% (238/1764)	15.6% (12/77)	17.2% (17/99)	0.84

Rates are mean ± SE. Cumulative outcomes use all oocyte retrieval cycles in each group as the denominator (one cycle per patient, integrating fresh and subsequent FET cycles); clinical pregnancy loss uses clinical pregnancies with a known outcome (live birth or confirmed loss) as the denominator, with outcome-unknown pregnancies excluded.

Pregnancies with an unknown outcome (ongoing pregnancy or loss to follow-up) were excluded from the pregnancy-loss denominator (168 in Non-PMOS, 9 in PMOS-nonIR, and 19 in PMOS-IR). Clinical pregnancy loss (no live birth among clinical pregnancies with a known outcome) showed a graded upward trend: 13.5% in Non-PMOS (238/1764), 15.6% in PMOS-nonIR (12/77), and 17.2% in PMOS-IR (17/99), but the between-group difference was not statistically significant (χ² test, *P* = 0.84). Owing to the limited number of loss events in the PMOS subgroups (n=17 and 12), this comparison should be interpreted with caution. Cumulative live birth rates did not differ significantly across the three groups (*P* = 0.7853). These findings indicate that IR-associated reproductive impairment manifests primarily at the oocyte-yield and transfer-opportunity level, while pregnancy loss showed only a non-significant upward trend.

## Discussion

In this study of 259 PMOS patients, we analyzed the dose–response relationship between continuous HOMA-IR and embryo development outcomes, retaining only the first PMOS cycle per patient to ensure statistical independence. The principal findings are as follows:

First, oocyte maturation rate showed no correlation with HOMA-IR (r=-0.014, *P* = 0.8178), unequivocally demonstrating that IR does not impair meiotic maturation.

Second, within the PMOS subgroup, HOMA-IR was negatively correlated with oocyte yield (r=-0.226, *P* = 0.0002). After adjusting for age, BMI, and AMH, each 1-unit increase in HOMA-IR was associated with approximately 0.605 fewer oocytes (β=-0.605, *P* = 0.0128). Combined with the finding that the PMOS-IR group required higher Gn doses yet yielded fewer oocytes, this result supports the hypothesis that IR reduces ovarian responsiveness to exogenous gonadotropins.

Third, once oocytes were retrieved, embryo developmental efficiency showed no association with HOMA-IR: transferable embryo rate r=0.042, *P* = 0.5082; blastocyst formation rate r=-0.036, *P* = 0.5673; usable blastocyst rate r=0.037, *P* = 0.5874. The normally cleaved to transferable conversion efficiency was nearly identical between groups (68.8% vs 67.4%), demonstrating that reduced oocyte yield — not impaired developmental efficiency — is the primary driver of the decline in absolute embryo numbers associated with IR.

Fourth, at the cumulative level, PMOS-IR patients had significantly higher cumulative β-hCG positive rate (85.4%) and cumulative clinical pregnancy rate (81.9%) than Non-PMOS controls (72.8% and 69.0%; *P* = 0.0024 and *P* = 0.0022). This advantage was driven primarily by greater transfer opportunity — 97.9% of PMOS-IR patients underwent at least one embryo transfer versus 89.0% of controls — whereas per-transfer implantation rates did not differ among groups, indicating that the between-group difference in cumulative pregnancy reflects differential transferable-embryo numbers (i.e., transfer opportunity) rather than per-transfer implantation efficiency. Concurrently, clinical pregnancy loss rose in a gradient across groups (17.2% in PMOS-IR, 15.6% in PMOS-nonIR, and 13.5% in Non-PMOS), but the between-group difference was not statistically significant. Cumulative live birth rates did not differ across groups (56.9% vs 56.5% vs 54.5%; *P* = 0.7853), indicating a limited net effect of IR on cumulative live birth.

The molecular basis of this pattern — high cumulative pregnancy alongside a trending increase in pregnancy loss — can be understood from two directions. On the one hand, hyperinsulinaemia may enhance endometrial receptivity by up-regulating epithelial IGF-1 signaling and GLUT4 expression (18, 19), and insulin itself exerts weak mitogenic activity that may promote early trophoblast proliferation. On the other hand, IR may impair endometrial decidualization and embryo implantation (21), while the accompanying systemic oxidative stress, mitochondrial dysfunction, and chronic low-grade inflammation (20, 22, 23) may compromise trophoblast invasion and spiral artery remodeling, leading to defective placentation — a mechanism supported by *in vitro* studies and gestational diabetes models (20, 24). That the pregnancy-loss trend did not reach statistical significance in this cohort suggests the clinical impact of these mechanisms on pregnancy maintenance is limited, or that the study was underpowered to detect it.

This pattern raises two clinically relevant questions. First, should luteal support be intensified for PMOS-IR patients? Given the non-significant between-group difference in pregnancy loss, the available evidence does not support intensified luteal support in this population — luteal support acts primarily on the endometrial receptivity window and has limited effect on trophoblast function (21); this awaits prospective evaluation. Second, should PMOS-IR patients be preferentially managed with a freeze-all strategy? In principle, supraphysiological oestradiol in fresh cycles may disturb endometrial–embryo crosstalk under IR, whereas FET endometrial preparation may provide a more favorable metabolic environment for implantation (25). To test this hypothesis directly, we compared pregnancy loss between fresh and frozen–thawed transfer cycles at the cycle level: overall, fresh-transfer loss was 19.2% (114/594) versus 22.9% (343/1499) for frozen–thawed transfer (*P* = 0.0689). By group, fresh versus frozen–thawed loss was 18.8% vs 22.7% in Non-PMOS (*P* = 0.0647), 11.1% vs 28.2% in PMOS-nonIR (*P* = 0.2203), and 31.0% vs 21.0% in PMOS-IR (*P* = 0.3122). Notably, frozen–thawed loss was no lower than fresh loss in the Non-PMOS and PMOS-nonIR groups, whereas the higher-fresh-than-FET trend in PMOS-IR rests on a limited number of events (only 9/29 fresh cycles with a known outcome) and did not reach statistical significance, most likely reflecting sampling fluctuation. Our data are therefore insufficient to recommend a universal freeze-all strategy for PMOS-IR patients, and larger studies are needed; the current strategy centered on embryo accumulation and individualized transfer remains appropriate.

Several features strengthen this study: analyzing HOMA-IR as a continuous variable preserved dose–response information; expressing embryo developmental efficiency as rates with appropriate denominators disentangled ovarian responsiveness from per-oocyte developmental competence; and retaining only the first PMOS cycle per patient ensured statistical independence.

Several limitations should be noted. First, the single-center retrospective design carries inherent selection bias, and the predominant use of GnRH-a long protocol with hCG trigger limits protocol and trigger variability, so the findings require multi-center validation. Second, between-group comparison of pregnancy loss was constrained by the limited number of loss events in the PMOS subgroups and should be interpreted with caution. Third, the absence of PGT-A data is a core limitation. Embryonic aneuploidy is the leading cause of IVF pregnancy loss, with approximately 50–70% of early miscarriages attributable to chromosomal numeric abnormalities (26). Recent evidence suggests that blastocyst aneuploidy rates differ between PMOS patients and controls (27). Hyperinsulinaemia under IR can induce oxidative stress and mitochondrial dysfunction in cumulus cells (28), which may theoretically increase the risk of meiotic spindle assembly errors and thereby elevate aneuploidy rates; however, the clinical value of PGT-A across PMOS phenotypes remains debated (29). If metabolic dysfunction does elevate embryonic aneuploidy, the upward trend in pregnancy loss observed across groups in this cohort may be driven in part by embryonic chromosomal factors rather than maternal endometrial factors — an unmeasured confounder that could influence the interpretation of the IR–pregnancy loss association, and one that this study cannot disentangle. Future studies should incorporate PGT-A data to clarify the relative contributions of maternal endometrial versus embryonic chromosomal factors to IR-associated pregnancy loss.

Future studies should validate the dose–response relationship observed here in prospective multicenter cohorts, incorporating PGT-A data and more comprehensive metabolic profiling (e.g., lipid profiles, SHBG) to develop more refined IR risk assessment models. Furthermore, whether insulin sensitizers such as metformin (12, 25) can ameliorate IR-related impairment of ovarian responsiveness warrants verification in RCTs.

## Conclusion and clinical implications

In this study of PMOS patients undergoing IVF, insulin resistance reduced ovarian responsiveness to exogenous gonadotropins in a dose-dependent manner, thereby constraining the absolute number of oocytes retrieved and, consequently, the number of transferable embryos. However, meiotic maturation, fertilization efficiency, and embryo developmental competence were preserved irrespective of IR status — a “quantity down, quality preserved” pattern.

These findings carry direct clinical implications: for PMOS-IR patients, the IVF bottleneck lies at the oocyte retrieval stage, not the embryo stage, and clinical strategies should prioritize optimizing ovarian stimulation to achieve adequate oocyte yield. Cumulative live birth rates did not differ across groups, indicating that the embryo quantity deficit associated with IR can be effectively compensated through FET; clinical pregnancy loss showed an upward trend without statistical significance, so close post-pregnancy surveillance and loss-risk assessment should be maintained for PMOS-IR patients.

HOMA-IR, as a routinely available metabolic marker, should be incorporated into pre-IVF ovarian response stratification for PMOS patients, but should not be used to predict embryo developmental competence. The non-significant upward trend in pregnancy loss warrants confirmation in larger prospective cohorts, together with investigation of the underlying mechanisms linking IR to oocyte yield. Randomized trials of insulin sensitizers to ameliorate IR-associated ovarian response impairment are warranted.

## Data Availability

The raw data supporting the conclusions of this article will be made available by the authors, without undue reservation.
