# Supplementary material for: Quantity down, quality preserved: a continuous HOMA-IR analysis of IVF/ICSI outcomes in PMOS patients
Source: Front Endocrinol (Lausanne). 2026 Sep 23;17:1930083. doi: 10.3389/fendo.2026.1930083 (PMC13642132; doi:10.3389/fendo.2026.1930083)
Supplement: Supplementary file 1 [file Table1.docx]

**Table S1. Spearman correlations (PMOS only, n=259).**

| **Variable** | **Spearman's r** | **_P_** | **Significance** |
| --- | --- | --- | --- |
| Oocyte count | -0.226 | 0.0002 | *** |
| MII oocyte count | -0.213 | 0.0006 | *** |
| Maturation rate | -0.014 | 0.8178 | ns |
| Fertilization rate | -0.105 | 0.0932 | ns |
| 2PN rate | -0.191 | 0.0021 | ** |
| Transferable embryo count | -0.252 | <0.0001 | *** |
| Transferable rate | 0.042 | 0.5082 | ns |
| Usable blastocyst count | -0.238 | 0.0001 | *** |
| Blastocyst formation rate | -0.036 | 0.5673 | ns |
| Usable blastocyst rate | 0.037 | 0.5874 | ns |

Note: ***_P_ < 0.001, **_P_ < 0.01, *_P_ < 0.05, ns: _P_ ≥ 0.05.
