# Supplementary material for: Quantity down, quality preserved: a continuous HOMA-IR analysis of IVF/ICSI outcomes in PMOS patients
Source: Front Endocrinol (Lausanne). 2026 Sep 23;17:1930083. doi: 10.3389/fendo.2026.1930083 (PMC13642132; doi:10.3389/fendo.2026.1930083)
Supplement: Supplementary file 2 [file Table2.docx]

**Table S2. Linear regression: HOMA-IR and embryo development outcomes (PMOS only).**

| **Dependent variable** | **Model** | **β (per HOMA-IR unit)** | **95%CI** | **_P_** | **n** | **R²** |
| --- | --- | --- | --- | --- | --- | --- |
| Transferable embryos | Unadjusted | -0.466 | -0.703–-0.23 | 0.0001 | 207 | 0.068 |
| Transferable embryos | Adjusted | -0.441 | -0.71–-0.172 | 0.0016 | 207 | 0.104 |
| Transferable rate | Unadjusted | 1.134 | -0.499–2.767 | 0.1751 | 207 | 0.009 |
| Transferable rate | Adjusted | 0.817 | -1.058–2.693 | 0.3940 | 207 | 0.033 |
| Usable blastocysts | Unadjusted | -0.294 | -0.456–-0.132 | 0.0005 | 207 | 0.058 |
| Usable blastocysts | Adjusted | -0.258 | -0.442–-0.073 | 0.0068 | 207 | 0.094 |

Note: Adjusted model includes age + BMI + AMH. Regression of HOMA-IR on oocyte count (adjusted β = −0.605, _P_ = 0.0128) is reported in the text. Transferable rate = transferable embryos / normally cleaved oocytes; its lack of association with HOMA-IR indicates that embryo developmental efficiency is independent of IR status.
